# Low-Cost Sensor Systems and IoT Technologies for Indoor Air Quality Monitoring: Instrumentation, Models, Implementation, and Perspectives for Validation

**DOI:** 10.3390/s25247567

**Published:** 2025-12-12

**Authors:** Sérgio Ivan Lopes, Cezary Orłowski, Pedro T. B. S. Branco, Kostas Karatzas, Guillermo Villena, John Saffell, Gonçalo Marques, Sofia I. V. Sousa, Fabian Lenartz, Benjamin Bergmans, Alessandro Bigi, Tamás Pflanzner, Mila Ródenas García

**Affiliations:** 1 ADiT-LAB—Applied Digital Transformation Laboratory, Polytechnic University of Viana Do Castelo, 4900-347 Viana Do Castelo, Portugal; 2 IBM Centre for Advanced Studies, Faculty of Computer Science and New Technologies, WSB University in Gdansk, 80-266 Gdansk, Poland; 3 LEPABE, ALiCE, Faculty of Engineering, University of Porto, Rua Dr. Roberto Frias, 4200-465 Porto, Portugal; pbranco@fe.up.pt (P.T.B.S.B.); sisousa@fe.up.pt (S.I.V.S.); 4 Environmental Informatics Research Group, School of Mechanical Engineering, Aristotle University of Thessaloniki, 54124 Thessaloniki, Greece; kkara@auth.gr; 5 Department of Physical and Theoretical Chemistry, Faculty of Mathematics and Natural Sciences, University of Wuppertal, 42097 Wuppertal, Germany; 6 NosmoTech Ltd., Cambridge CB3 0AZ, UK; 7 Polytechnic Institute of Coimbra, Technology and Management School of Oliveira Do Hospital, Rua General Santos Costa, 3400-124 Oliveira do Hospital, Portugal; 8 ISSEP—Institut Scientifique de Service Public, 4000 Liege, Belgiumb.bergmans@issep.be (B.B.); 9 Department of Engineering ‘Enzo Ferrari’, University of Modena and Reggio Emilia, 41125 Modena, Italy; alessandro.bigi@unimore.it; 10 Department of Software Engineering, University of Szeged, 6725 Szeged, Hungary; 11 EUPHORE Labs., Fundación Centro de Estudios Ambientales Del Mediterráneo (CEAM), 46980 Paterna, Valencia, Spain

**Keywords:** low-cost sensors, indoor air pollution, sensor systems, IoT, remote monitoring

## Abstract

**Highlights:**

**What are the main findings?**

- A system-level framework is presented that links low-cost indoor air quality (IAQ) sensors (EC, MOS, NDIR, PID, optical) with IoT technologies (e.g., LoRaWAN, NB-IoT, MQTT) and field calibration approaches (co-location and ML-based on-field corrections) to achieve reliable indoor measurements.
- The paper consolidates validation and certification practices—including key performance indicators (KPIs), management of drift and cross-sensitivity, mitigation of sampling losses, and data synchronisation techniques—into a practical roadmap spanning steps from laboratory characterisation to in situ field verification.

**What is the implication of the main finding?**

- This work provides actionable guidance for practitioners on instrumentation selection, IoT-based telemetry design, and the implementation of robust QA/QC procedures (including uncertainty assessments, standardised data formats, and systematic maintenance protocols) for achieving scalable and reliable IAQ monitoring in buildings.
- Supports regulators and standards bodies by outlining harmonised procedures that improve trust, comparability, and readiness for the certification of low-cost IAQ sensor systems.

**Abstract:**

In recent decades, significant efforts have been devoted to constructing energy-efficient buildings, providing comfortable indoor environments. However, measures such as enhanced airtightness, while reducing infiltration through the building envelope, might consequently reduce natural ventilation. This reduction is a critical concern because natural ventilation is an essential factor in controlling indoor air quality (IAQ), and its diminution could therefore worsen IAQ. Sick building syndrome has emerged as a term used to describe health hazards linked to the time spent indoors but with no particular cause. Since people spend most of their time indoors, the demand for continuous and real-time IAQ management to reduce human exposure to pollutants has increased considerably. In this context, low-cost sensors (LCS) for IAQ monitoring have become popular, driven by recent technological advancements and increased awareness regarding indoor air pollution and its negative health impacts. Although LCS do not meet the performance requirements of reference and regulatory equipment, they provide informative measurements, offering high-resolution monitoring, emission source identification, exposure mitigation, real-time IAQ assessment, and energy efficiency management. This perspective article proposes a general model for LCS systems (and subsystems) implementation and presents a prospective analysis of their strengths and limitations for IAQ management, reviews the literature regarding sensor system technologies, and offers design recommendations. It provides valuable insights for researchers and practitioners in the field of IAQ and discusses future trends.

## 1. Introduction

Humans are exposed to air pollutants both outdoors and indoors. Although we spend about 90% of our time at home or in other indoor environments [1], indoor air quality (IAQ) has not received adequate attention. Complex mixtures of chemicals present indoors, which may be emitted intermittently or continuously, can adversely affect both IAQ and human health [2]. Indoor environments encompass a heterogeneous range of infrastructures, including schools, hospitals, homes, fitness centres, offices, and public transport systems. Each of these are potentially characterised by distinct pollutant sources.

In the building sector, high energy-efficient (HEE) design has gained increasing attention in recent years. One of the key strategies to achieve HEE buildings is improving airtightness, which often leads to a reduction in natural ventilation. Ventilation enables air exchange between indoor and outdoor environments and is essential for controlling IAQ and reducing the transmission of airborne bacteria and viruses [3]. As airtightness increases, building occupants may experience Sick Building Syndrome (SBS) [4]. This condition describes situations where individuals experience health issues associated with time spent indoors, without a clearly identifiable cause.

Given that most of our time is spent indoors, there is a growing need for continuous, real-time IAQ monitoring to manage exposure to pollutants in poor-quality environments. Building Management Systems (BMS), such as Heating, Ventilation, and Air Conditioning (HVAC) and Smart Home Systems (SHS), are now integral to new building designs. These systems pave the way for the development of “cognitive buildings” that surpass conventional automation by integrating cognitive computing techniques. Such approaches analyse and interpret building data, operational procedures, and occupancy patterns to deliver actionable insights for more effective IAQ management.

At the physical layer, this vision relies on pervasive sensing, achievable only by reducing the cost of IAQ sensing elements and systems while increasing their deployment density indoors. Consequently, the development of Low-Cost Sensors (LCS) and Systems (LCSS) for IAQ has gained significant momentum due to advances in Microelectromechanical Systems (MEMS) [5,6], the Internet of Things (IoT) [7,8], and Artificial Intelligence [9,10]. These LCSS have also received growing attention owing to increased awareness of indoor air pollution and its impact on human health.

Although LCS often fall short of the performance of regulatory-grade and reference instruments, they can still provide valuable measurements. This enables high-resolution monitoring, source identification, exposure assessment, real-time IAQ management, and energy efficiency optimisation [11,12]. In its 2021 report, the World Meteorological Organization [13] highlighted the current and potential roles of LCSS in multiple applications involving IAQ monitoring:

1. Research in Atmospheric Sciences: Sensor systems (SS) are beginning to play a role in areas such as model and emissions validation, as well as in the study of spatial variability in air pollution.
2. Long-Term Global Change: SS are increasingly being used as complementary information sources to well-established reference monitoring systems.
3. Air Quality Management: This represents one of the earliest application areas for SS, supporting both operational and research-driven air quality assessment.
4. Air Quality Compliance and Regulation: It remains unlikely that SS will be adopted for regulatory purposes unless standardised protocols are developed for calibration, operation, and data utilisation.
5. Public Information and Community Monitoring: This category already includes several active applications of SS, enabling citizen science and local engagement in air quality awareness.
6. Proxy for Exposure Assessment: An emerging application area where SS are increasingly replacing low time-resolution passive sampling methods for personal and environmental exposure evaluation.

Most limitations of LCSS are associated with sensor accuracy. Although some sensors are considered inadequate for outdoor air applications, several of them can still provide satisfactory data quality for indoor environments. This is because the concentration levels of pollutants differ substantially between indoor and outdoor air. Moreover, indoor conditions such as temperature and humidity are generally more stable and constrained.

The measurement characteristics of a sensor system—namely its sensitivity, resolution, span, time response, and sampling rate—depend on the intended application. Typically, when a target value (TV) exists, an LCSS should demonstrate (1) a sensitivity and resolution such that the lowest measurable concentration is smaller than the TV; (2) a span greater than the TV; and (3) a response time and sampling interval shorter than the averaging interval of the TV.

For common IAQ applications, the World Health Organization (WHO) indoor air quality guidelines [14] provide such target values, which can serve as a basis for protecting public health in exposure studies. More recent recommendations by the French Agency for Food, Environmental and Occupational Health & Safety [15] offer additional or alternative target values in similar contexts. Other target values have been proposed by various international and national organisations, including those established in European and national legislation.

Given the multidisciplinary nature of this field—which encompasses analytical chemistry, microelectromechanical systems (MEMS), electronics, instrumentation, computer science, and data analytics—it is essential to establish a common understanding of the current state of the art. Such an approach promotes a shared conceptual and technical foundation among researchers and engineers regarding the terminology and paradigms to be considered in the design and evaluation of LCS for IAQ management.

The selection of studies was done in a way that considered relevance to indoor air quality sensors, technological scope, and methodological quality, identified and guided by the author’s expertise and experience in the field. This selection was supported by keyword-based searches in major databases (Scopus and Web of Science) resulting in a number of primary papers and reviews. These provided a broad, robust and balanced overview on different aspects. In this work, the authors have identified, synthesised and structured the key knowledge and concepts essential for understanding and advancing the topic of LCSS.

This article proposes a general model for the implementation of LCS systems and subsystems, offering a prospective analysis of their strengths and limitations for IAQ management. It provides an overview of SS technologies, includes design recommendations, and discusses future trends. This paper is intended for a broad audience, particularly engineers and developers seeking to design IAQ monitoring devices based on LCS. It should also serve as a useful reference for: (1) Indoor air scientists, building managers, and practitioners seeking to deepen their understanding of sensor system technologies and platforms; and (2) Public health professionals, policymakers, and citizens wishing to become familiar with state-of-the-art low-cost sensor technologies for exposure assessment applications.

## 2. Generic Sensor System Model

A generic sensor system model that provides a common ground throughout this document will be presented. This paper will focus on the core terms coined in the literature, namely sensor and sensor system. It will not address other common and trendy terms that have been introduced in recent decades, such as smart sensor, smart transducer, intelligent sensor, and intelligent transducer, because these terms include, by definition, a sensor or a sensor system as subsystems. For example, the term “smart sensor” was introduced in [16], and the “smart” prefix has since been used for two main distinctions: (1) a sensor that performs a function that could not be previously performed; or (2) a low-cost sensor implementation that was previously not economically viable. Moreover, the terms “smart” and “intelligent” have been widely used to show technological improvements and are also being commercially exploited to promote consumer-type products. Due to these ambiguous usages, the authors opted, in the present paper, to focus only on the generic model of a sensor system including its core associated technologies. These are organised by hardware and software, and describe some application case examples.

Throughout this document, the definitions presented in [17] will be adopted, where

- Sensor element: This is the fundamental transduction mechanism (e.g., a material) that converts one form of energy into another. Some sensors may incorporate more than one sensor element (e.g., a compound sensor).
- Sensor: A sensor element including its physical packaging and external connections (e.g., electrical or optical).
- Sensor system (SS): A sensor and its assorted signal-processing hardware (analogue or digital), with the processing either in or on the same package or discrete from the sensor itself.

**Figure 1:**
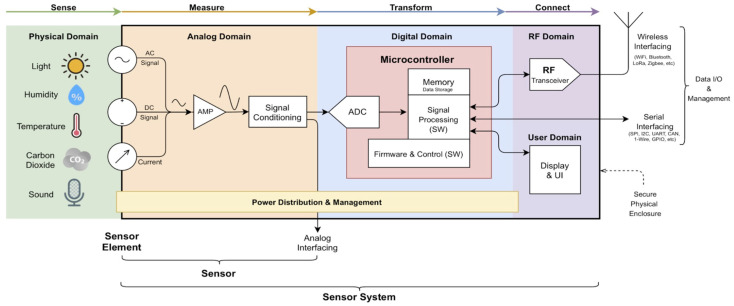
Generic Sensor System Model: A Schematic Representation.

A sensor model is an abstract representation of the physical sensing device along with its data processing and communication mechanisms, which can be divided into three main sub-components [18]: (1) the observation model, which describes a sensor’s measurement characteristics; (2) the dependency model, which describes a sensor’s dependence on other external sources; and (3) the state model, which describes how a sensor’s observations are affected by its location and internal state. At this point, the authors aim to propose a generic sensor system model (cf. Figure 1) and will not consider the sub-components previously introduced, since they are specific to each sensor or sensor system implementation.

The physical enclosure or housing is a relevant component in a sensor system. Depending on the application, it may include weatherproofing, electromagnetic shielding, and mounting. However, these site-specific requirements for housing may incur additional costs for the final device. Despite being mandatory in outdoor applications, weatherproofing is usually unnecessary in indoor applications and thus not considered to reduce costs. On the other hand, other issues are important to consider for the physical enclosure or housing. These include Electromagnetic Compatibility (EMC) protection, protection from insects, shielding from thermal radiation, and adequate ventilation for the sensors. EMC shielding protection is relevant to protect a sensitive signal from external electromagnetic signals or to prevent a stronger signal from leaking out and interfering with surrounding electronics. In Europe, it is normally specified as meeting the Radio Equipment Directive 2014/53/EU and EMC Directive 2014/30/EU. Enclosures in IAQ applications rarely suffer from EMC emission problems but fail on susceptibility. It is also important to ensure that housing protects sensors from insects that may enter and become trapped, leading to false readings or even damage to sensors. This protection is normally achieved by installing a stainless-steel or polypropylene (PP) mesh on the inlets and outlets, which also prevents other coarser particles from blocking the sensor. In some cases, a fluoropolymer mesh may be needed for gas sampling inlets. While shielding from thermal radiation in SS for outdoor applications involves a Stevenson screen or similar, indoor environments require simpler protection. Here, where solar radiation is much lower, the enclosure usually only requires adequate ventilation and protection from direct sunlight and other radiant sources. Last but not least, it is important to provide adequate ventilation to the sensors, either by diffusion or assisted by a fan. The enclosure should be designed to ensure proper airflow to all sensors.

## 3. Sensor Technologies

A sensor includes a sensor element (transducer) and converts the received signal into an electrical form of signal (voltage, current, etc.). Prior to analogue-to-digital conversion, most analogue signals require conditioning, such as signal scaling (division, amplification, and shifting), filtering, or isolation (e.g., DC component removal). These steps ensure a compatible voltage range before digitisation. In the following sub-sections, sensor technologies used in the implementation of LCS will be introduced, focusing on their operating principles. Table 1 presents a comparative analysis of the examined sensor technologies.

### 3.1. Electrochemical Sensors (EC)

Nowadays, it is common to find electrochemical sensors in LCSS. They are used for the detection of a wide variety of gases of environmental importance, including carbon monoxide (CO), carbon dioxide (CO_2_), nitrogen dioxide (NO_2_), and ozone (O_3_). The term “electrochemical sensor” includes conductimetric, chemiresistive, potentiometric, and amperometric sensors [19]. Amperometric gas sensors generate a current by reaction (oxidation or reduction) of the analyte on a catalytic electrode [20]. Standard amperometric electrochemical sensors consist of three components: a gas chamber, an electrochemical cell, and a reservoir. The gas chamber controls gas access and hence sensor sensitivity, and adding a chemical filter can improve gas selectivity. The electrochemical cell itself consists of a working electrode, a counter electrode, and usually a reference electrode [21]. A fourth auxiliary electrode may be included to compensate for baseline changes in the sensor. The electrodes are saturated with an electrolyte solution. The electrolyte reservoir accommodates changes in equilibrium electrolyte concentration as the relative humidity (RH) changes [22]. Indeed, temperature and RH are the dominant factors affecting EC sensor performance, especially under changing conditions. They must be accounted for in their calibration and data analysis [22,23]. Recent studies have shown that modifications in electrode material can improve selectivity, stability, and lower detection limits [24].

### 3.2. Metal Oxide Semiconductors (MOS)

The operating principle of Metal Oxide Semiconductors (MOS) is based on the absorption of oxygen at high temperatures at the surface of the metal oxides. More specifically, there are three regions of the semiconductor: the surface, which interacts with the gas; the bulk, which remains unaffected; and the particle boundary, located between these two regions [25]. Resistance is affected since, at high temperatures, oxygen atoms bond onto the boundary, extracting electrons in the process from the semiconductor’s surface region for n-type MOS. The oxygen then either directly reacts with ambient gases, or these gases also bond onto the sensor, which causes more charge carriers to be withdrawn or injected into the surface region [26]. The semiconductor (n or p) type determines the way resistance is affected by gas interactions. In p-type semiconductors, where holes are the majority charge carriers, oxygen atom reactions reduce the resistance. Reducing gases have the opposite effect for a p-type semiconductor [26,27]. SnO_2_, an n-type semiconductor, is the most common MOS sensing material. Its electrical resistance decreases when it is in contact with the target gas. This resistance can be highly sensitive to the type and concentration of different gases [28,29]. MOS sensors can be easily manufactured and implemented and can yield a response from a few seconds to minutes [30]. However, n-type MOS suffer from cross-sensitivity with humidity, attributed to the interaction of hydroxyl ions with the oxide surface [31]. MOS sensors show sensitivity to a wide range of inorganic gases such as CO, sulphur dioxide (SO_2_), hydrogen sulphide (H_2_S), and ammonia (NH_3_). A variety of sensing materials are typically used to measure gases, e.g., ZnO, SnO_2_, and TiO_2_ for CO, and WO_3_ for O_3_ and NO_2_ sensors [32].

### 3.3. Photoionization Detectors (PID)

**Figure 2:**
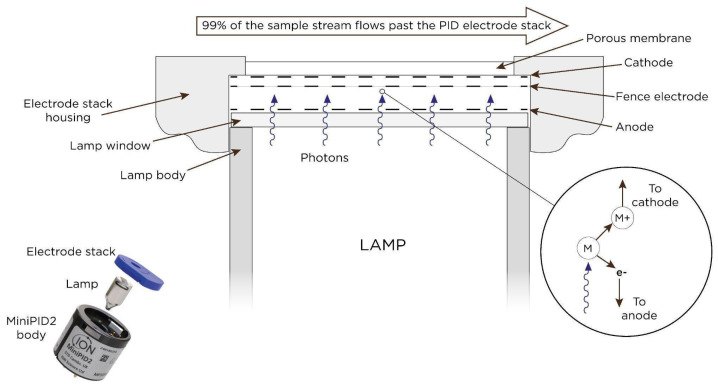
PID–type sensor schematic (courtesy Ion Science Ltd., Fowlmere, UK).

A photoionisation detector (PID) is based on the absorption of photons (hv) from a photodischarge source by a molecule of the target gas, leading to the formation of an excited molecule, i.e., photoions. When hv is higher than the ionisation energy of the molecule, it emits an electron and a cation is formed. The cation is driven to an electrode, generating an electrical current that is a function of the number of excited molecules and therefore of the concentration of the gas sensed [33]. Several types of lamps are employed in PIDs. Krypton lamps emit light at 123.9 and 116.9 nm (10.0 and 10.6 eV). Argon lamps emit at 105.9 nm (11.7 eV), while xenon lamps emit at 147.6 and 129.1 nm (8.4 and 9.6 eV). PIDs are only selective by the ionisation eV and cannot distinguish between different VOCs because any molecule with an ionisation energy lower than the photon energy of the lamp will contribute to the signal. For this reason, isobutylene is commonly used as a reference calibration gas and specific response factors are applied to evaluate the readings for other VOCs [34]. However, some lamps offer better sensitivity. For example, the 10.0 eV (Krypton lamp with CaF_2_ filter) gives good sensitivity to BTEX VOCs with reasonable selectivity. The 11.7 eV lamps ionise most VOCs, while the 10.6 eV Krypton lamp with an MgF_2_ filter is the most popular PID lamp with a high intensity and long lamp lifetime (>5000 h). PIDs respond rapidly (t_90_ = few seconds), and their minimum detection limit can be as low as 100 ppt. They also exhibit a very stable baseline. Figure 2 presents a schematic representation of PID operation. Typically, PIDs are commonly provided in a standard form factor of 20 mm diameter and 16.6 mm height.

### 3.4. Non-Dispersive Infrared (NDIR)

Non-dispersive infrared (NDIR) technology relies on passing infrared radiation through a sample and determining the fraction of the incident radiation that is absorbed at a particular wavelength. NDIR can be considered a special case of infrared (IR) absorption spectrometers, where only transmission for single or several selected wavelengths is recorded to identify gases. The NDIR sensor consists of a polychromatic light source (lamp or LED), gas cell, band-pass filter, optical window, and detector. The band-pass filter is positioned in front of the detector to restrict the incoming light to the specific wavelength band associated with a target analyte, which enhances the selectivity of the sensor. There are many pollutant gases that absorb in the mid-IR (3–7 μm) region, including CO, CO_2_, SO_2_, nitrogen oxides (NOx), nitrous oxide (N_2_O), NH_3_, hydrogen chloride (HCl), hydrogen fluoride (HF), and methane (CH_4_). Quantification of NDIR measurements is based on the Beer–Lambert Law, where the light absorbed by the gas sample is proportional to its concentration [35,36]. NDIR is less prone than other technologies (e.g., electrochemical) to poisoning and drift. It also exhibits high precision, accuracy, selectivity, and long service life compared to other methods [37].

The three major drawbacks of NDIR sensors are power requirements, minimum detection limit, and interference. The detection limit is influenced by the intensity of the IR source, the design of the path chamber, and the detector. Improvements in these components have led to better accuracy and sensitivity [38]. Interferences may occur when other gases absorb at the same wavelengths. For example, water vapour shows significant absorption peaks in the 2 to 8 μm spectral region. Narrow band-pass filters are used to limit the detection of the analyte gas to wavelengths where there is no interfering absorption. Still, water vapour-related drawbacks can remain and need to be addressed [39].

Different optical gas cell designs are used, with an important difference being the use of single- or dual-path optics. In the latter, the second reference channel is tuned to a wavelength that is not affected by the parent gas concentration [40]. This allows the correction of the measurement in the main channel with the reference signal. As a result, the system compensates for drift in the IR light source, changes in temperature and pressure in the chamber, temperature drift of the detector, and changes in the optical path (e.g., dirt on the optics). The outcome is a better baseline and long-term stability.

Recently, there has been an upsurge of NDIR CO_2_ sensors on the market related to the characterisation of ventilation rate, occupancy, and air quality. Main improvements in NDIR sensor technology have been applied to this type of sensor. Typically, CO_2_ LCS report accuracies of either ±30 ppm or ±50 ppm (plus ±3% or ±5% of the measured value). This accuracy is acceptable for most indoor applications, such as evaluation of room occupancy levels and ventilation rates. Photoacoustic sensors are an equivalent technology, relying on the pressure wave generated when a molecule absorbs the IR photon. In this case, a simple microphone replaces the photodiode as a detector. The advantage is a lower price and a smaller package, but sensitivity to vibrations and other acoustic interferences can be a problem.

The next generation of NDIR sensors is now becoming available, using LEDs and photovoltaic detectors. These devices consume very low power (0.5 to 2 mW RMS power vs. 100 to 450 mW for tungsten lamps). The result is a CO_2_ NDIR sensor that can be battery-powered for months. They also generate less heat, allowing faster stabilisation when switched on. An added benefit is that when the LED is off, there is no radiation. In contrast, a tungsten lamp continues emitting for seconds after switch-off. The LED NDIR therefore has a repeatable off-signal, giving better baseline stability. However, tungsten NDIR sensors have the advantage of self-heating by typically 5 °C, avoiding condensation on the optics at high humidities.

### 3.5. Light Scattering

When monitoring aerosols, the most common detection principle is measuring the intensity of scattered light by a single particle, usually termed Optical Particle Counters (OPC) [41], or an ensemble of particles (nephelometric scattering), depending on the optical arrangement. In both cases, the air sample is pulled due to a pressure gradient caused by a fan, a pump, or a heating element, into the sensing volume, where the light scattered by the aerosol is received by a photodetector. The resulting signal is then amplified and filtered, finally producing an analogue voltage or a pulse-width modulation signal.

Single-particle detection uses a collimated light beam that is condensed into a small sampling volume. The photodetector pulses from each particle are collected and used to derive the particle number concentration (PNC). The pulse height is used to derive the size of each particle, which is subsequently assigned to tabulated size bins (generally ranging between 6 and 24 size bins, depending on the low-cost OPC model). LCS OPCs generally detect size ranges between ~300 nm and 40 µm [42], depending on the quality of the optical and electronic components and the optical cell design. The number size distribution is used to calculate the particulate mass concentration (PM), assuming a spherical particle shape and estimating the refractive index and particle density.

When sampling larger volumes, the light is scattered by an ensemble of particles and detected either across a wide angular range (e.g., ~10–170°) or at a single angle. Accordingly, these particle monitors are referred to as nephelometers and photometers [41]. Several photometers and nephelometers report low-pulse occupancy (LPO), i.e., the percentage of time when the light path from the source to the phototransistor contains particles. Other instruments instead report an analogue voltage [43]. These latter outputs are generally calibrated to PM_2.5_ levels and, depending on the sensor, they might provide multiple aerosol size ranges, e.g., both PM_10_ and PM_2.5_. There are about 50 different sensors marketed for aerosol detection based on light scattering [42], which are used in many applications.

This technique depends not only on the instrumental setup but also on the chemical composition of the aerosol, its water content, shape, and size. Unlike OPCs, these instruments do not measure particle number concentration directly. Therefore, they cannot provide information on the particle size distribution. Another disadvantage is that they are normally calibrated for measuring PM_2.5_ and have difficulty measuring particles larger than 3 µm. As a result, PM_10_ is frequently calculated from PM_2.5_ and not measured directly.

Optical monitors, such as OPCs, photometers, or nephelometers, all suffer at high relative humidity values (i.e., RH > 60%). Under such conditions, particles grow by water adsorption, producing larger particle sizes. Heaters can be used to dry particles, but with the ensuing large power requirement. They are also limited by wavelength when measuring small particles, meaning that most optical PM monitors cannot measure particles smaller than 300 nm.

### 3.6. Ionisation Chambers

An ionisation chamber is an active sensor element often used for detecting ionising radiation. In the case of radon, they are used for detecting alpha particles and can operate for short-term and long-term assessments. An ionisation chamber establishes an electric field between two or more electrodes, allowing filtered air to diffuse into the chamber. If a charged or ionised particle passes through the chamber, electron–ion pairs are generated and accelerated due to the applied electric field until they collide with the electrodes [44]. The electronic circuit generates an electric pulse for each collision, which is then counted over specific time intervals [45]. The measured ionisation is caused by the decay of radon and its decay products. Two measurement modes are possible: either the total ionisation in the chamber is measured, or the pulses caused by different individual alpha particles are counted separately. The latter approach allows distinguishing between pulses caused by different decay products (e.g., polonium-214) and by radon. The response time depends on the rejection of counts from late decay products and on the rate of replacement of air in the ionisation chamber. Although ionisation chambers are usually able to operate over a wide range of concentrations, temperatures, and humidity, the presence of thoron (another uranium decay product) can affect accuracy. Thoron can contribute up to 10% in general environments, but it can be more significant in thoron-enhanced areas [45]. Typically, LCS radon implementations use ionisation chambers [46,47] due to their simple production, affordability, and satisfactory accuracy. It is important to note that widely used passive and integrating techniques, such as electret-based devices [48] are excellent for determining time-averaged concentrations but fall outside the scope of this work, which focuses on active, real-time systems.

### 3.7. Alpha Spectrometry

Alpha spectrometry is a well-established technique for radionuclide assay and environmental radiation monitoring, particularly for detecting radon (Rn-222) and its short-lived progeny in air. The method relies on measuring the energy of alpha particles emitted during radioactive decay, which provides a direct way to identify specific isotopes and quantify their activity. It was demonstrated that alpha particles separated measurements can be achieved through track size distribution [49]. In radon monitoring, alpha spectrometry typically employs solid-state detectors—most commonly silicon semiconductor devices—capable of resolving the characteristic energy peaks of radon progeny such as Po-218 and Po-214 [50]. This energy discrimination enables accurate conversion of count rates into concentration values.

Applications of alpha spectrometry in indoor air sensing include real-time radon monitoring in residential environments, workplaces, and calibration laboratories. Several studies have demonstrated its use in portable systems for environmental surveys and building diagnostics [51,52,53]. Recent research has explored low-cost implementations using silicon PIN photodiodes, which are sensitive to alpha particles and can be integrated with commercial off-the-shelf electronics for compact sensor designs [54,55,56]. These developments aim to make active radon detection more accessible for large-scale indoor air quality monitoring.

The main advantages of alpha spectrometry include its high sensitivity and ability to discriminate between different alpha-emitting isotopes, allowing precise radon concentration measurements even in mixed environments. Unlike passive methods such as alpha-track detectors or electret ion chambers, alpha spectrometry provides near real-time data and current technologies can distinguish radon from thoron when required. However, the technique also presents limitations: the calibration is complex, as detectors cannot be individually calibrated for all alpha emitters, and environmental factors such as humidity and dust can affect collection efficiency [50].

Despite these challenges, alpha spectrometry remains one of the active methods for radon detection and continues to evolve toward more affordable and portable designs. Its integration into low-cost sensor platforms represents a promising direction for improving indoor air quality assessment.

## 4. Sensor Systems Technologies

Contemporary sensor systems (SS) are a key element in the development of modern measurement technologies, automation, and the Internet of Things (IoT). They support the collection, processing, and analysis of environmental data in real time, enabling precise decision-making and process optimisation. Advances in miniaturisation, wireless communication, and energy-efficient electronics have significantly increased their functionality and availability.

SS extend the abilities of sensors—as introduced in the previous section—by adding additional features and possibilities. Typically, SS have one or more integrated sensors that handle sensing/transduction, amplification, and signal conditioning. In addition, they also include parts for analogue-to-digital conversion, digital signal processing, data integrity check, data transmission, data display, and, in specific cases, the possibility for remote device management (cf. Figure 1) [57,58]. The combination of these features is normally driven by specific application requirements. For example, a SS can be designed to include a user interface (display, pushbuttons, knobs, etc.) and/or to include external connectivity (wired or wireless). In some application domains, the physical attributes of the SS may be of great importance. Therefore, other relevant requirements may be put forward, which can include remote manageability, robust physical enclosure including weatherproof characteristics, and the provision of security mechanisms (in both physical and cyber domains). In the next subsections, the digital domain implementation will be described and discussed (cf. Figure 1), with a focus on analogue sensor interfacing, common computational systems, and signal processing with an emphasis on sensor characteristic linearisation.

### 4.1. Hardware Architecture and Signal Conditioning

The hardware architecture of sensor systems dictates the basis of their operation, determining how measurement data is acquired, processed, and transmitted. A key element of this architecture is the signal chain, which includes both sensors and signal amplification and filtering systems. The signal conditioning process aims to improve data quality by eliminating interference and adapting electrical parameters to the requirements of analogue-to-digital converters. Common signal conditioning circuits include amplification, filtering, and impedance coupling [59]. Recent advances in the design of integrated circuits (ICs) allow the implementation of sensor signal conditioner (SSC) ICs with very high-quality and reliable specifications [60,61]. Each type of sensor requires appropriate signal conditioning, from simple Resistor-Capacitor (RC) filters (the basic building block of simple passive filters used in signal conditioning), to sophisticated instrumentation amplifier circuits. Conditioning also includes voltage stabilisation, temperature compensation, electronic noise suppression, and factory or field calibration [62,63]. The importance of repeatability and drift in LCS is very high, which, without a proper analogue path, can significantly distort data.

### 4.2. Analogue Interfacing

The analogue interface is a key element of electronic systems, enabling the processing of real-world signals. It allows the conversion of physical quantities such as temperature, pressure, or light intensity into electrical signals understandable by digital systems. Using appropriate analogue circuits enables filtering, amplification, and conditioning of input signals. Analogue sensor outputs (voltage, current, etc.) are normally converted to digital quantities (e.g., digital code) to simplify processing, storage, and data transmission. This conversion is performed by an analogue-to-digital converter (ADC), which is an electronic component designed to operate within a voltage range for a specific resolution in bits. This conversion process is composed of three main steps: (1) sampling, where the input voltage is stabilised and then sampled using a sample-and-hold electronic circuit; (2) quantisation, which represents the approximation (for a specific resolution in bits) of the input voltage to obtain the digital output code; and (3) codification, which contemplates the final preparation of the digital data before interfacing with a microprocessor, e.g., data parallelisation or serialisation [64]. LCS implementations typically use low-cost ADCs that require calibration routines. The total error resulting from the analogue-to-digital conversion refers not only to the quantisation error but also to offset and gain errors that need to be compensated. Moreover, there are also non-linearities that result from the manufacturing process that may have a relationship with temperature, frequency, and supply voltage operation [65]. These dependencies may be an important source of error that must be compensated for through software calibration routines to achieve higher accuracy.

### 4.3. Computational Systems and Data Interfacing

Computing systems in the realm of IoT are basic elements enabling the integration of peripherals, network infrastructure, and data processing layers, and processors are their core element. Common processors used in the design of IoT devices include the microcomputer and the microcontroller unit (MCU). However, these terms are often misused and represent distinct general-purpose computational resources that may be used in different application domains. Microcomputers are computing devices that include a microprocessor, a single-chip implementation normally referred to as the central processing unit (CPU), programme and data memory, interrupt handling blocks, and I/O interfacing. On the other hand, the MCU is a low-cost system-on-chip that includes a CPU and several general-purpose built-in modules. These modules typically include analogue-to-digital converters (ADC), data and programme memory (EEPROM, FLASH, RAM), clock generation and distribution, power management, and multiple digital interfacing ports (USART/UART, SPI, I^2^C, CAN, 1-Wire, GPIO, PWM, etc.), which enable multiple possibilities for external communications, among other peripherals. IAQ LCS implementation typically relies on the use of MCUs or microcomputers, mainly due to their affordable price and general-purpose architectures. Digital sensor communication normally uses I^2^C, SPI, or UART. Furthermore, the maturity of the IoT ecosystem, its low cost, its ubiquity, and interoperability-driven models have been key drivers for the successful development of several hardware platforms that are being used not only in research and academia but also commercially.

### 4.4. Preprocessing and Data Aggregation in Sensor Systems

In sensor systems, data preprocessing and transmission play a key role in ensuring the efficient operation of the entire measurement infrastructure. Preprocessing reduces the amount of raw data through filtering, aggregation, and compression of information directly at the sensor nodes. This approach lowers energy consumption, reduces the load on the communication network, and increases transmission reliability. Therefore, the appropriate selection of transmission methods and communication protocols is a factor to be taken into account, as it influences the performance and durability of sensor systems.

Coming to the sensor element itself, computational power can be used to process and transform the digital version of the acquired signal(s). A crucial step in this process is related to sensor characteristic linearisation and ad hoc calibration procedures. At this point, the available computational power can be used to apply: (1) signal processing techniques [66], e.g., sensor characteristic linearisation [67] and signal denoising [68]; (2) data analytics methods, e.g., for sensor data quality assessment [69,70]; and (3) machine learning techniques, e.g., for field calibration [71]. For example, digital signal processing techniques are commonly used in SS for sensor characteristic linearisation. In this case, the sensor’s output signal is determined by its characteristic, which describes the relationship between the measured quantity and the output signal. If the characteristic is time-invariant, a transfer function can be obtained. However, non-linear sensor outputs are often linearised for ease of design and calibration and to improve measurement accuracy. The accuracy of linearisation can be impacted by the difficulty of finding a function that matches the sensor response curve precisely. While analogue methods are still popular due to the availability of high-performance analogue devices, digital methods offer several advantages. When implemented with software and supported by back-end servers or dedicated microcontrollers, digital techniques can offer increased accuracy and flexibility with reduced time and cost requirements. However, they also require higher processing power and memory, which can limit their effectiveness [67].

## 5. Indoor Air Quality Remote Sensing Platforms

Modern indoor environmental monitoring systems increasingly utilise remote measurement platforms, enabling continuous, real-time air quality assessment. The development of IoT technologies and the miniaturisation of sensors have enabled the integration of various measurement modules with cloud platforms, significantly increasing data availability and precision. Such solutions play a key role in analysing parameters such as carbon dioxide concentration, volatile organic compounds, humidity, and temperature. As a result, remote measurement platforms are an important element of modern air quality management systems in the building sector.

To develop a network of indoor air pollution sensors, it is essential to select appropriate devices and communication protocols for data processing. Therefore, having architectural knowledge of remote sensing platforms becomes necessary to explain the importance of using suitable technology to measure indoor air quality [72]. This section is divided into three main parts. Firstly, the Web and IoT stack is mapped to the TCP/IP reference model (Table 2). Secondly, the development of IoT platforms is presented, including a brief description of the individual layers of this architecture used for IAQ assessment [73]. Thirdly, the architecture of IoT systems that extend classic sensory platforms is discussed. In addition, individual layers of this architecture are discussed, along with relevant examples of IoT use for developing remote sensing platforms. Finally, relevant examples of IAQ building management platforms, described in accordance with the adopted architectural approach, are presented. The use of a coherent architectural description significantly enhances the organisation of the examples presented for the use of sensory platforms in IAQ assessment [74,75]. Additionally, it serves as an introduction to the certification processes outlined in the following chapter.

**Table 3:** Overview of the main network layer protocols and their specifications within the IoT protocol stack.

Protocol	Operating Frequency	Maximum Range	Throughput	Latency	Reference
802.15.4-Based (Zigbee, WirelessHAR)	900 MHz, 2.4 GHz	~200 m	250 kbps	10–100 ms	[76]
NB-IoT (LTE Cat NB2)	Cellular bands	1–10 km	159 kbps	1.6–10 s (NB1)	[77]
LTE-M2 (LTE Cat M2)	Cellular bands	>11 km (M1)	4 Mbps (DL), 7 Mbps (UL)	10–15 ms (M1)	[77]
Sigfox	868 MHz, 902 MHz	>50 km (M1)	100–600 bps	-	[78]
Bluetooth 5	2.4 GHz, 5 GHz	<200 m (PIP), <1.5 km (mesh)	1 to 3 Mbps	<3 ms	[79]
LoRaWAN (LoRa)	915 MHz (US), 868 MHz (Eur), 433 MHz (Asia)	5–20 km	0.3–50 kbps	-	[80,81]
Wifi (802.11 a/b/g/n)	2.4, 3.6, 4.9, 5, 5.9 GHz	<100 m	>54 Mbps	1–3 ms	[82]
Z-Wave	868.42 MHz	<100 m	100 kbit/s	1–3 ms	[83]
Thread	2.4 GHz	<30 m	250 kbps	-	[84]

### 5.1. Overview and Classification of Telemetry Platforms

Telemetry platforms are a component of monitoring and data analysis systems, enabling remote measurement, transmission, and interpretation of environmental, technical, and biological parameters. Numerous attempts are made in [85] to classify these platforms, taking into account application criteria, data transmission types, and degree of integration with analytical systems. The purpose of this section is to provide an overview of contemporary telemetry platforms and organise them according to selected classification criteria and reference models.

An integral component of telemetry platforms is communication. The TCP/IP reference model is a four-layer stack commonly used to model communication networks and associated communication protocols. It provides a general framework that can be mapped to different stacks such as the classic Web Stack or newer IoT Stack (cf. Table 2). Networks based on IoT and sensor networks are understood as networks composed of multiple devices deployed in a specific area to perform a common task [37]. Communication protocols are defined as a set of rules and actions that devices follow when establishing communication and exchanging data [86].

Given the scope of this work and the stacks presented in Table 2, the IoT stack will be briefly introduced. The application layer commonly uses the following protocols:

- Representational State Transfer (REST): A style of software architecture derived from the experience of writing HTTP specifications for distributed systems. REST describes a machine-to-machine interface and allows content to be rendered on demand.
- Constrained Application Protocol (CoAP): An IoT application protocol for resource-constrained devices based on REST, which allows the use of a client-server model to communicate with devices identified by URIs [87]. The CoAP protocol aims to address the heavy demands on HTTP resources [88]. It has lower overhead and only uses a 4-byte fixed header. CoAP operates over UDP (User Datagram Protocol) and can be secured using DTLS (Datagram Transport Layer Security) for encryption. However, due to its reliance on UDP, this protocol inherits messaging unreliability and can experience issues with NAT (Network Address Translation) traversal.
- Message Queuing Telemetry Transport Protocol (MQTT): A lightweight, publish/subscribe messaging protocol ideal for IoT applications, constrained devices, and unreliable networks. Its minimal overhead, robust connection handling, and flexible Quality of Service (QoS) levels make it efficient and scalable for connecting a large number of devices while ensuring reliable data transfer [89]. Other protocols similar to MQTT include the Extensible Messaging and Presence Protocol (XMPP), an open set of technologies for instant messaging, and the Advanced Message Queuing Protocol (AMQP), developed for message-oriented middleware. AMQP offers strong reliability with QoS but is resource-intensive, making it suitable mainly for applications with high security requirements.

The transport layer uses the TCP (Transmission Control Protocol) and UDP protocols as the basic transport layer protocols for data transfer on the user-server network line [72]. However, each of these protocols works differently and is therefore used for different purposes. Compared to TCP, the operation of UDP is simpler and faster. In the UDT protocol, data is streamed continuously, without splitting into sequences, acknowledgements or error correction. UDP streaming does not stop when the data packet is lost. Therefore, it is worth considering the use of the UDT protocol in IAQ measurement networks.

The internet layer includes the following protocols: IPv6, 6LoWPAN, and RPL. IPv6 (Internet Protocol Version 6) is the successor to IPv4, developed from the depletion of IPv4 addresses [90]. The purpose of this version of the protocol is to increase the number of available addresses, to organise the protocol header, and to introduce extensions. IPsec (Internet Protocol Security, IP Security) is a set of protocols used to implement secure connections and exchange encryption keys between computers. These protocols can be used to create a Virtual Private Network (VPN), which may be important from the point of view of building IAQ measurement systems [91]. The IoT network layer, as described in Table 2, will be introduced and discussed in the next section.

### 5.2. Internet of Things Protocol Stack

The IoT relies on a complex ecosystem of communication protocols that enable efficient data exchange between devices with limited resources and diverse architectures. The IoT protocol stack is a multi-layered framework integrating technologies for transmission, routing, security, and device management in distributed networks. Unlike the classic TCP/IP model, it is optimised to minimise energy consumption, reduce bandwidth, and reduce communication latency. Analysing the individual layers of the IoT protocol stack allows us to understand how various standards—such as MQTT, CoAP, and 6LoWPAN—interact to ensure the interoperability and reliability of IoT systems.

Low-Power Wide Area Networks (LPWANs) describe a broad class of radio communication technologies (including LTE-M, NB-IoT, Sigfox, and LoRaWAN) [92] that are used for long-distance wireless communication [93]. Their main advantage is ultra-low power consumption, which allows relevant sensors to work for up to 10 years on a single battery. This makes them a very interesting communication technology for small IoT objects in indoor environments. LPWANs also have a very wide range, allowing wireless sensors to transmit digital information over long distances (up to 30 km in an outdoor environment) and are much less expensive to access and deploy [94]. However, their biggest problem is the limited bandwidth compared to other radio technologies (data throughput is typically between 100 bps and 10 kbps), which makes this technology unsuitable if a large amount of continuous data exchange is expected.

On the other hand, Personal Area Networks (PAN) are a type of network that can be used to connect electronic devices over short distances within a person’s working area. Typically, PANs are used to connect objects such as smartphones, tablets, or IoT objects to a host device acting as a gateway (i.e., a computer connected to the Internet). PANs can be wireless (WPAN, i.e., Wi-Fi, Bluetooth, ZigBee) or carried over wired interfaces such as USB [95]. A very high speed of data transfer is possible, but the power consumption is high and, in practice, requires the operation of the connected object in sectors.

Another common type of IoT network is the Body Area Network (BAN), most used in healthcare IoT applications [96]. The security requirements of these applications are critical. The last group of measurement networks is mesh networks. This is a type of network where measurement nodes form a network, and individual nodes communicate with each other. Within this network, there is also a wired network that connects only selected nodes. This is an example of a highly reliable network resulting from the possibility of creating any internal network [97].

The IoT Stack network layer is a group of many architectural solutions and requires a broader explanation. Table 3 lists and describes the main IoT networking protocols commonly used in IoT Stack implementations.

### 5.3. Edge–Fog–Cloud Processing Architecture

The concept of IoT involves the ubiquitous behaviour of cyber–physical systems with increased sensing capabilities. These systems can be identified and managed with a unique address [90] and collaborate in real time to achieve common goals. IoT architectures utilising information and communication technologies (ICT) have been explored to improve public health and well-being [98] and productivity in daily activities [99]. Real-time monitoring of IAQ is essential in most residential environments [100]. Data collected by IoT systems can alert residents on IAQ and assist physicians in diagnosis related to health [101]. Smart Building is an effective IoT application domain related to IAQ. They integrate elements such as objects, sensors, and actuators that communicate and interact to remotely enhance building management functions [92]. BMS can automate control and automation tasks like HVAC, lighting, and security. The use of LCS and IoT technologies to enhance BMS capabilities is critical to offset the energy efficiency of buildings with IAQ [102].

**Figure 3:**
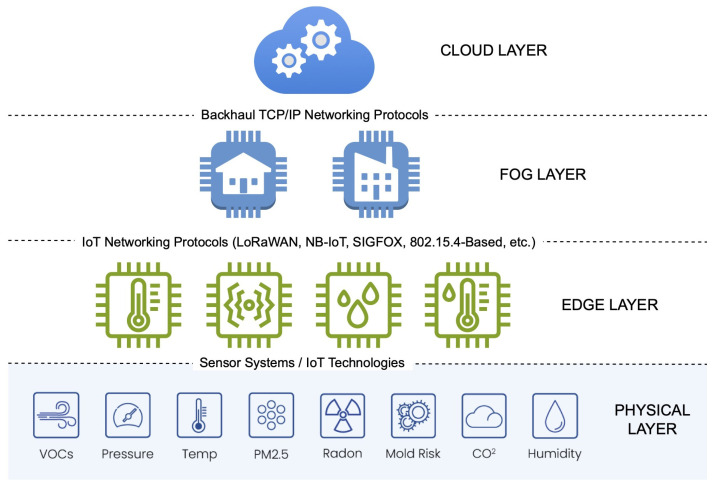
IoT Edge–Fog–Cloud Layered Architecture.

IoT architectures require specialised data processing mechanisms due to the vast amounts of data involved [93]. These mechanisms are typically mapped to the classic TCP/IP communication model, which divides IoT-based architectures into three core layers (Figure 3): Edge, Fog, and Cloud [103,104,105].

The IoT Edge–Fog–Cloud computing architecture is an integrated computing model that combines computing and storage resources distributed across different layers of the network infrastructure. It enables efficient processing of data generated by IoT devices, by pre-analysing it at the edge and fog levels, followed by further processing in the cloud. This approach reduces latency, optimises bandwidth utilisation, and increases the security and reliability of distributed systems.

The Edge layer, also known as the data provider layer, is the foundational layer in IoT networks. This layer is responsible for acquiring data, which may be pre-processed and then transferred to higher layers [94]. It is where IoT nodes, equipped with microcomputers and sensor elements, are configured; therefore, they may represent IAQ sensor node capabilities of the network. The number of nodes and their connection method are determined by the network topology. In the three-tier model used for data processing, the way in which the Edge layer is built, data is processed, and subsequently transferred to the Cloud, is dependent on the functionality of these networks. For IAQ remote sensing, nodes can be placed in different environments, such as buses or buildings [72]. In addition, the number of nodes, the way these nodes are built, and the adopted data processing techniques depend on the network size and the specific requirements of the application of interest.

On the other hand, the Fog layer is similar to the previously introduced Edge layer but typically has more processing power, which is the basis of preprocessing tasks [89]. This is where the data collected are queued, in many cases using the MQTT protocol. This layer is the basis for data enrichment using data mining processes, or it is also a source of pre-processed data for the Cloud layer. The structure of the Fog layer is based on both clusters of virtual machines, very often single virtual machines, and applications created for preprocessing tasks, functioning as a key layer in the IoT model [95].

The Cloud layer works like a typical layer found in IT systems. It is here that the computing power is greatest, and this is where data with a high degree of certainty go, which is the basis for building training and test sets for forecasting based on machine learning [106]. It is worth noting that in IoT models, this is the layer that is only used when the preprocessing processes on the Fog layer do not provide network functionality. The goal is to keep processing as low as possible. This means that if we can make predictions at the Fog layer level, we should do so. Therefore, as mentioned above, the data collected in this layer are the basis for the use of artificial intelligence mechanisms such as fuzzy modelling, artificial neural networks, or genetic algorithms. Each layer of the Edge–Fog–Cloud model uses the IoT Stack protocols presented in Table 3.

### 5.4. Implementation Examples and Use Cases

This section presents selected examples of the implementation of such systems and discusses their practical applications in various types of residential and non-residential buildings.

- Smart home air quality monitors—These systems utilise multiple sensors (to measure CO_2_, PM_2.5_, volatile organic compounds (VOCs), humidity, and temperature) to assess indoor air quality in real time. They connect to mobile apps or smart home assistants (such as Alexa or Google Home), allowing users to track air quality trends and receive alerts about rising air pollution levels [37,106].
- Building management systems (BMS) with air quality sensors—These systems, in office buildings and public buildings, integrate air quality sensors with the building management system (BMS). They continuously measure parameters such as CO_2_ levels, temperature, and humidity, and automatically adjust ventilation or HVAC systems to maintain optimal air quality and energy efficiency [107].
- Industrial indoor air monitoring systems are systems used in factories, laboratories, and cleanrooms to ensure compliance with occupational health and safety standards. These systems monitor particulate matter (PM_2.5_, PM_10_), gases (CO, NO_2_, O_3_), and volatile compounds, generating detailed data logs for analysis and regulatory reporting [108].
- Healthcare facility monitoring systems in hospitals and clinics utilise advanced IAQ systems to ensure a clean and safe environment for patients and staff. Sensors detect CO_2_, PM, and airborne pathogens, and integrated control systems optimise ventilation and filtration based on real-time data [101,109].

An IoT-based system for IAQ that includes a dust sensor, temperature and humidity sensor, and provides an Air Quality Index (AQI) value based on Raspberry Pi was presented by Shaheen et al. [110]. A real-time standalone air quality monitoring system that includes PM_2.5_, CO, CO_2_, temperature, humidity, and air pressure was also proposed by Kumar et al. [111]. This system combines the computational resources of a Raspberry Pi with the compatibility of sensors available for Arduino. The sensorial unit is interfaced with an Arduino UNO, connected by USB to the Raspberry Pi, which acts as a gateway for data sharing. An IoT-based IAQ monitoring system based on Raspberry Pi that incorporates temperature, humidity, and an MQ-5 gas sensor was studied by Kiruthika et al. [112]. The proposed system also provides data sharing through Twitter.

In Taiwan, the Environmental Protection Administration uses an air quality monitoring network that people can access; however, this data only shows large areas, and there is no way to know the real situation in individual houses. A more practical solution is to monitor real-time concentration data while observing air quality based on the architecture of a smart home system. The Arduino Uno board, ESP8266 wireless transmission technology, and various sensors were used as the basic hardware, and the desktop terminal software was developed with standard programming tools. According to the results of the experiment, indoor environment data were analysed, and the load was controlled by combining fuzzy logic rules in the layer [73].

Lastly, Yasin et al. [90] present the development of an IoT system for monitoring IAQ. The main elements of the edge layer used in the physical circuit are the Arduino Leonardo, dust sensor, temperature and humidity sensor, LCD display, and fan. The interactive platforms involved are The Things Network for Fog for middleware development. The data detected by the physical circuit are converted into an Air Quality Index (AQI), which is then sent to an interactive cloud platform. In [93], the key decisions made by researchers and building managers who want to carry out their own environmental monitoring study using commercially available hardware and software are outlined. The collected data can be easily integrated with open-source analytics software to visualise and make informed decisions about IAQ management in the cloud.

### 5.5. AI/ML for Indoor Air Quality Remote Sensing Platforms

AI/ML solutions for indoor air quality measurement are emerging as an extension of the outdoor air quality monitoring network paradigm, as well as due to the proliferation of smart sensors and indoor environment automation in modern buildings.

The role of AI/ML in indoor air quality sensor platforms is related to aspects such as (a) data pre-processing and analysis (harmonisation, handling missing data and outliers, correlation analysis, etc.), (b) improving the performance of individual sensors and sensor networks, (c) increasing the explainability of sensor behaviour and its relationship to other indoor environmental parameters (such as energy consumption, light levels, etc.), and (d) supporting decision-making, particularly through Large Language Models (LLM) and others [113,114]. These aspects can be handled by different layers of the telemetry system: lightweight computations can be performed at the edge, while more demanding processes can be handled in the cloud. An important aspect of utilising different layers is their ability to support data security and protect data integrity, which is a prerequisite for open and interoperable indoor air quality monitoring systems.

GenAI then brings additional value to remote indoor air quality (IAQ) monitoring platforms. This value can be implemented in four areas:

- Data acquisition and calibration: language models can simulate data from various scenarios, including automatic data calibration based on patterns from large environmental datasets [115].
- Analysis and enrichment of sensor signals: this involves data fusion, creating conditions for generating environmental documents [114].
- Creation of air quality predictions and simulations: language models can support predicted IAQ changes over the short and medium term (e.g., within a few hours or a day) [116].
- Support for decision-making processes: language models can generate recommendations for, for example, ventilation systems. Support for Building Management Systems is also important, allowing for the automatic control of HVAC, air purifiers, and humidifiers [107].

### 5.6. Synthesis of Development Prospects for IAQ Telemetry Platforms

The development of telemetry platforms dedicated to IAQ analysis is a significant trend in environmental engineering and smart building management systems. Contemporary solutions of this type combine the acquisition, processing, and visualisation of data from distributed sensor networks, monitoring microclimate parameters. By integrating sensors with telemetry platforms it is possible to collect and analyse data in real-time using ML algorithms. This integration supports the process of optimising environmental conditions in enclosed spaces while establishing robust situation awareness capabilities that inform dynamic IAQ management requirements. Situation awareness in IAQ systems refers to the continuous perception and comprehension of environmental parameters, their relationships, and their projected future states. This framework is essential for developing responsive IAQ management strategies that adapt to changing occupancy patterns, external pollution conditions, and operational demands.

Another key trend is the implementation of cloud architectures, enabling scalable and flexible processing of measurement information while maintaining the data integrity necessary for long-term environmental compliance monitoring. In the context of further development, telemetry platforms analysing IAQ are increasingly utilising edge computing technologies and AI mechanisms to increase operational efficiency. Moving some analytical calculations to local measurement nodes reduces transmission delays and improves system responsiveness. The use of predictive algorithms enables the identification of pollution trends and the forecasting of changes in microclimate parameters, taking into account operational and environmental factors. As a result, these platforms perform not only monitoring functions but also diagnostics and support decisions regarding ventilation management, air filtration, and building energy efficiency.

The incorporation of blockchain technologies into IAQ telemetry platforms represents another emerging frontier in ensuring data authenticity and maintaining immutable records of environmental measurements. The technology has already been tested for ambient air monitoring demonstrating its advantages [117]. Blockchain-based architectures enable decentralised validation of AQ sensor data, creating transparent audit trails that support regulatory compliance and facilitate multi-stakeholder verification. Furthermore, distributed ledger systems provide resilience against single points of failure, ensuring continuity of situation awareness even during localised infrastructure disruptions. The immutability of blockchain records proves particularly valuable for facilities subject to stringent environmental health and safety regulations, as it establishes verifiable provenance for all IAQ measurements and corrective actions undertaken.

The development of telemetry platforms for IAQ analysis focuses on increasing interoperability, data security, and compliance with international environmental standards. Increasing importance is being placed on the implementation of communication protocols based on open standards, such as MQTT and LoRaWAN, as well as the use of cryptographic mechanisms to ensure the integrity and confidentiality of transmitted data. In the medium and long term, the development of these platforms will be strongly linked to the concept of smart buildings and sustainable development strategies. Within this framework, telemetry data forms the basis for automatic control systems for indoor environments. A synthesis of these trends indicates that air quality analysis platforms will play an increasingly important role in improving the comfort, health, and energy efficiency of public spaces.

## 6. Validation, Certification, and Calibration

### 6.1. Validation and Certification

Sensor system networks provide vast amounts of data, which could improve the spatiotemporal resolution necessary when mapping IAQ, monitoring pollutants to compare with limit values, providing data for IAQ models, or locating pollution sources. Ensuring good data quality remains a challenge. Determining data quality has, until recently, been addressed by publishing statistical analyses of either field trials or laboratory testing, without following any prescribed test procedures or data analysis. Each commercially available SS should be laboratory-tested for performance over a range of temperatures and humidity, with attention to interferences and short- and long-term stability. Field trials are necessary because controlled laboratory tests do not replicate in-field variability. Many studies report poorer indicators of performance in real-world conditions than in laboratory tests (AQ-SPEC).

Certification and testing (C&T) are important operations towards establishing LCS as appropriate for IAQ estimations. Despite the lack of a regulatory framework, there have been various initiatives to develop a structured and repeatable LCS testing process.

The most consistent source for validating single SS has been the Air Quality Sensor Performance Evaluation Center (AQ-SPEC), operated by the South Coast Air Quality Management District in Los Angeles. However, these tests are limited to field (outdoor) trials in Southern California referenced to Federal Reference Methods (FRM) and Federal Equivalent Methods (FEM) certified instrumentation, thus within a hot, semi-arid climate (Köppen–BSh classification). Standards committees are now introducing Test Specifications (TS) for validating SS performance and providing classification according to the expanded uncertainty at limit values. The first TS published for LCS was a CEN standard [118], focusing on the performance evaluation of air quality SS for gaseous pollutants. The second TS that followed was a CEN standard [119] for particulate matter in ambient air.

These standards provide detailed procedures for C&T of LCS, and state the criteria according to which such SS may be classified, as follows:

- Class 1 sensor system: sensors at minimum consistent with indicative methods;
- Class 2 sensor system: sensors at minimum consistent with objective estimation methods;
- Class 3 sensor system: sensors not formally associated with any mandatory target.

This certification allows for the categorisation of sensors according to the relative expanded uncertainty of the measurement, which varies per pollutant, as defined in the relevant annex of the Air Quality Framework Directive (2008/50/EC). Even if this document focuses only on ambient outdoor air, it remains the most important initiative to standardise testing procedures and requirements for LCS, and part of the reported results are also useful to assess LCS intended for indoor use. The American Society for Testing and Materials (ASTM) D22.03 (Work Item WK 64899) is a draft for a similar TS for SSs. Meanwhile, a new standard test method has been published for evaluating indoor PM_2.5_ by the American Society for Testing and Materials [21]. This standard was developed in light of the emerging needs of the indoor air ventilation industry, in collaboration with the AQ-SPEC programme of the USA South Coast area (http://www.aqmd.gov/home).

### 6.2. Lab and Field Calibration Methods

Most LCS suffer from cross-sensitivity with other contaminants and dependence on temperature and relative humidity. In most cases, these performance characteristics are quantified in the manufacturer’s specifications of the LCS. Nevertheless, calibration with a reference instrument must be performed prior to their installation in the field. Linear Regression (LR) models and the Multilinear Regression (MLR) model are the most widely used techniques to calibrate low-cost sensor data against a reference measurement [43]. However, machine learning methods hold great promise. Unfortunately, the calibration of sensors in laboratory settings fails to fully encompass the extensive range of conditions present in indoor environments, largely due to the complex nature of the field trials. While laboratory or simulation chamber tests are appropriate for examining cross-sensitivities with various gaseous species [120] and the influence of relative humidity and temperature, they do not account for the full range of gases and volatile organic compounds (VOCs) present indoors, nor do they capture the dynamics of aerosol concentrations and their associated physical and optical properties. These factors are recognised as sources of error for LCS measurements. Therefore, on the one hand, it is recommended to also calibrate sensors in an indoor environment, under conditions as close as possible to those where the measurements will take place. On the other hand, the application of analysis models successfully used in calibrations outdoors should be approached with caution when used indoors, given the different number and types of contaminants in both environments [11].

Additionally, any SS must be recalibrated periodically. The calibration interval depends on the design of the SS, the manufacturer’s recommended calibration period, and any regulatory or user-defined accuracy requirements or guidelines. Calibration interval is of course directly linked to the purpose of the measurement and accepted uncertainty, but on a general basis, if an optical sensor can be recalibrated on a yearly basis or more, it is recommended to check electrochemical sensors twice a year and MOX sensors even on a more regular basis. It is a good practice to establish an SS or SS network in the required location and allow it to be measured for one or two weeks before final pre-campaign calibration. This reduces the uncertainty due to the environmental conditions being different from the initial laboratory calibration or earlier calibration in a different location. Periodic calibration of the SS and validation of data quality can be achieved by six methods, as follows.

The “Golden Sample” method is becoming increasingly popular for SS calibration. An additional SS that is regularly calibrated at a reference station or laboratory is termed a “Golden Sensor System”. It is circulated between SS sites for comparison. Unfortunately, this method depends on the same sensor technology, so bias errors that are inherent in the technology (e.g., RH transients with electrochemical gas sensors) may go unnoticed. This procedure should be carefully organised to ensure the accurate traceability of the Golden Sensor System.

Another calibration method is periodic removal and then co-locating all the SSs in the network next to a reference station. This method essentially returns the network to the “as-installed” condition but involves the difficult logistics of removing and redeploying the entire SS network, plus the necessary downtime of the network.

Co-location with a reference station of at least one SS in a network for local continuous calibration by co-location is the most popular method for SS or SS outdoor network calibration but is not always possible indoors. The reference station should be as close as possible to ensure that both the SS and reference station sample air under the same conditions. Algorithms that use co-location to determine drift across the entire network are used by some manufacturers and researchers to extrapolate calibration at a single point to baseline calibration across the network.

A fourth method applies calibrated gas mixtures to each SS on-site, in situ, but delivering accurate gas concentrations is unwieldy, and providing known concentrations of particles and their particle size distributions is even more difficult.

A fifth method is to use a mobile reference station that moves between each SS for a short co-location period (typically 1 to 7 days). This calibration method, although reliable, is difficult to achieve indoors due to logistical problems of remote powering and environmental control for most reference and equivalent instrumentation.

Therefore, while laboratory tests are suited for evaluating cross-sensitivities, field calibration using periodic co-location within different indoor environments or fixed reference nodes remains the most reliable approach for ensuring data comparability across deployments. Nevertheless, for long-term or varying condition monitoring indoors, where although environmental parameters are relatively stable, contaminants can be very heterogeneous and sensor drift may occur, the use of adaptive or model-assisted calibration techniques can be more efficient. In this context, machine learning calibration methods offer a promising complement to conventional field procedures. Overall, the calibration strategy should reflect the monitoring purpose: co-location for controlled or comparative studies, and introduction of model-assisted calibration techniques when flexibility and adaptation to varying conditions are required. The latter approach will be discussed in the next subsection.

### 6.3. Machine Learning-Computational Intelligence-Based On-Field Calibration

Machine learning methods can be used to model the behaviour of LCS. Thus, LCS measurements are used as input to the modelling procedure, together with other data coming from the LCS system (typically measurements of additional pollutants as well as indoor environmental conditions such as temperature and relative humidity). Then, a model is developed, trained, tested, and validated, resulting in an output that is as close as possible to the ground truth [11]. This procedure has been shown to improve not only the basic statistical indices of sensor performance (R, R^2^, RMSE, MAE, etc.) but also to drastically reduce the relative expanded uncertainty of the measurement, thereby rendering LCS appropriate for uses not previously foreseen (i.e., as complementary measurements to official instruments for outdoor environments, according to the CAFE Directive for urban air quality) [71].

Several machine learning methods have been widely used to enhance sensor performance, with most of them applied for ambient air monitoring. For example, Random Forests were applied to low-cost sensor data to resolve the spatial heterogeneity in air contaminants [121]. Moreover, simple linear regression, multiple linear regression, neural networks, and XGBoost were used, with neural networks presenting the best results in terms of RMSE [122]. In addition, ensemble learning, input optimisation, the use of machine learning methods like stacking, and the combined use of meta-learners proved to be valuable additions to the efforts made towards individual as well as sensor network calibration [123]. Research from ambient air studies denoted three main challenges for machine learning calibration: the need for a massive number of data points to be collected, the existence of missing data, and the fact that calibration models need to be sensor-specific [122] —the latter being gradually addressed by recent works [124].

Concerning indoor environments, machine learning models for calibration purposes have also been used. The Gaussian Process Regression method was tested for low-cost PM sensor calibration in Patra et al. [125]. The authors collected data on air temperature, humidity, HVAC operation, indoor combustion sources, and cooking practices, and the results successfully validated the use of machine learning-based calibration methods. The work presented in Chojer et al. [12] achieved high accuracy with on-field (nurseries and primary schools) calibration boosting (GBR and XGB) and SVR models for PM (PM_1_, PM_2.5_, and PM_10_) sensors, after also applying multiple linear regression. Both of these studies concluded that the proposed models are site-specific, which remains a challenge in the application of machine learning calibration. On this basis, network-oriented, on-the-fly calibration can be investigated to allow for a generalised calibration procedure applicable to a whole network of LCS rather than individual sensors. In Bagkis et al. [123] it was demonstrated how genetic algorithms can be used in parallel with hybrid stacking to employ a combination of batch machine learning algorithms and regularly updated online machine learning calibration functions for the whole network, provided that a few reference instruments are present. Although their approach was demonstrated for ambient air, it is agnostic to environmental conditions and may therefore be considered suitable for indoor air as well.

Random Forest regression model training using co-located sensors was also successfully applied in Bush et al. [126]. Indeed, due to their low cost, sensors can be duplicated to allow drift or other problem correction. It should also be mentioned that, in the calibration of LCS and SS for long-term IAQ monitoring, it is necessary to account for sensor performance degradation [127].

It is relevant to mention the importance of collecting all the environmental factors that impact the quality of the sensor-collected data. However, the inclusion of a broad number of features increases the complexity of the model. Therefore, feature selection mechanisms are critical to identifying the best combination of features to be used [128]. Interestingly, the same seems to be the case in the use of machine learning methods for IAQ prediction [113,129,130,131,132].

A comprehensive review concerning LCS calibration and its applications is included in a recently published study [133], where ML has been identified as the most advanced method for dynamic sensor calibration under real-world operational conditions.

Overall, the results presented in this work have shown that the use of machine learning techniques for low-cost calibration presents promising results. However, several limitations still need to be addressed by future research.

## 7. Conclusions

This work addresses the gap left by the absence of comprehensive reviews that integrate all aspects of sensor system implementation. It begins by identifying the key components required for the development of Indoor Air Quality (IAQ) monitoring devices based on Low-Cost Sensors (LCS). The study defines the relevance of LCS as the foundation of a generic sensor system model and provides a detailed explanation of its core elements. This paper serves as the basis for a holistic overview of the design and implementation of sensor systems (SS) built around LCS technologies. The model is subsequently expanded through an analysis of its main building blocks—sensor technologies, computational platforms, and connectivity solutions. These are examined to highlight their interdependence in bridging sensing activities with Internet-based functionalities. Furthermore, the work discusses validation, calibration, and certification procedures, offering insights into key methodologies and emerging practices. By critically evaluating the strengths and limitations of each element, this review provides valuable guidance for the practical and scientific application of LCS in IAQ monitoring.

This review integrates the multidisciplinary aspects of IAQ monitoring using LCS within a unified conceptual framework. Structuring the information around a sensor system model enhances the understanding of the underlying technologies, their interactions, and their overall system-level behaviour. Consequently, this work can serve as a reference document for researchers, health professionals, practitioners, building designers, policymakers, educators, and end users. It facilitates the design of more efficient and targeted IAQ monitoring strategies and fosters a clearer understanding of the potential and limitations of LCS-based systems.

Beyond synthesising current approaches, this study highlights future research needs in calibration and validation, particularly the integration of machine learning techniques to improve accuracy, stability, and compensation for cross-sensitivities. Progress is also needed towards the standardisation and certification of LCS data to ensure comparability, reliability, and transparency, enabling regulatory acceptance of these technologies. Advancements in pollutant-specific sensing, especially for volatile organic compounds (VOCs), remain essential to enhance selectivity and precision. Additionally, the growing integration of artificial intelligence into LCS systems opens new possibilities for automated data correction, predictive analytics, and adaptive IAQ management.

Finally, the continued development of LCS technologies requires close interdisciplinary collaboration among engineers, architects, health scientists, computer scientists, and policymakers. Such cooperation is vital to translate technological progress into reliable, evidence-based IAQ strategies and to establish LCS as a mature, credible, and widely accepted technology for environmental monitoring.

## Figures and Tables

**Table 1:** Comparative analysis of the examined sensor technologies.

Sensor Technology	Indoor Air Pollutants	Approx. Accuracy	Response Time (t_90_)	Key Interferences & Influences	Relative Cost
Electrochemical (EC)	CO, NO_2_, O_3_	Varies by gas and calibration	Seconds to minutes	Temperature, Relative Humidity, Cross-sensitive gases (e.g., O_3_ for NO_2_)	Low
Metal Oxide (MOS)	CO, NO_2_, NH_3_, O_3_, H_2_S	Qualitative / Trend-based	Seconds to minutes	Humidity (major), Temperature, Broad cross-sensitivity	Low
Photoionization (PID)	VOCs (e.g., BTEX)	Depends on calibration gas (e.g., isobutylene)	Few seconds	Any VOC with Ionisation Potential < lamp energy; Cannot distinguish between VOCs	Medium- High
Non-Dispersive Infrared (NDIR)	CO_2_	±30 ppm or ±50 ppm (±3–5% of reading)	Seconds to minutes	Water vapour	Medium- High
Light Scattering (OPC)	PM_2.5_, PM_10_	Varies; calibrated for PM_2.5_	Real-time (minutes for mass conc.)	Particle composition, Refractive index, High Humidity (>60%)	Low- Medium
Ionization Chamber	Radon (Rn-222)	Satisfactory for LCS (e.g., ±15–20%)	Hours (due to air replacement & decay product rejection)	Thoron (can contribute ~10%)	Medium
Alpha Spectrometry	Radon (Rn-222)	High sensitivity and precision	Near real-time (minutes to hours)	Humidity, Dust, Complex calibration	High

**Table 2:** Web and IoT protocol stacks consist of layered communication architectures and technologies that enable data exchange between devices, servers, and applications within the Internet and the Internet of Things (IoT) environments.

Layers	Web Stack	IoT Stack
Application Layer	Web Applications	IoT Applications, Device Management
	HTTP, DHCP, DNS, TLS/SSL	REST, COAP, MQTT, XMPP, AMQP
Transport Layer	TCP, UDP	TCP, UDP
Internet Layer	IPv6, IPv4, IPsec	IPV6, 6LOWPAN, RPL
Network/ Link Layer	Ethernet (IEEE 802.3), DSL, ISDN, Wireless LAN (IEEE 802.11), Wi-Fi	IoT network protocols (to be described in Table 3)
